# Long-term efficacy and safety of osilodrostat in patients with Cushing’s disease: results from the LINC 4 study extension

**DOI:** 10.3389/fendo.2023.1236465

**Published:** 2023-08-23

**Authors:** Mônica Gadelha, Peter J. Snyder, Przemysław Witek, Marie Bex, Zhanna Belaya, Adina F. Turcu, Richard A. Feelders, Anthony P. Heaney, Michaela Paul, Alberto M. Pedroncelli, Richard J. Auchus

**Affiliations:** ^1^ Neuroendocrinology Research Center, Endocrinology Section, Medical School and Hospital Universitário Clementino Fraga Filho, Universidade Federal do Rio de Janeiro, Rio de Janeiro, Brazil; ^2^ Perelman School of Medicine, University of Pennsylvania, Philadelphia, PA, United States; ^3^ Department of Internal Medicine, Endocrinology and Diabetes, Medical University of Warsaw, Warsaw, Poland; ^4^ Department of Endocrinology, University Hospitals Leuven, Leuven, Belgium; ^5^ Department of Neuroendocrinology and Bone Disease, Endocrinology Research Centre, Moscow, Russia; ^6^ Division of Metabolism, Endocrinology and Diabetes, University of Michigan, Ann Arbor, MI, United States; ^7^ Department of Internal Medicine, Endocrine Section, Erasmus Medical Center, Rotterdam, Netherlands; ^8^ Division of Endocrinology, Diabetes and Metabolism, Department of Medicine, David Geffen School of Medicine, University of California, Los Angeles, Los Angeles, CA, United States; ^9^ Novartis Pharma AG, Basel, Switzerland; ^10^ Recordati AG, Basel, Switzerland; ^11^ Department of Pharmacology, University of Michigan, Ann Arbor, MI, United States

**Keywords:** Cushing’s disease, osilodrostat, hypercortisolism, 11β-hydroxylase, long-term treatment

## Abstract

**Objective:**

To evaluate the long-term efficacy and safety of osilodrostat in patients with Cushing’s disease.

**Methods:**

The multicenter, 48-week, Phase III LINC 4 clinical trial had an optional extension period that was initially intended to continue to week 96. Patients could continue in the extension until a managed-access program or alternative treatment became available locally, or until a protocol amendment was approved at their site that specified that patients should come for an end-of-treatment visit within 4 weeks or by week 96, whichever occurred first. Study outcomes assessed in the extension included: mean urinary free cortisol (mUFC) response rates; changes in mUFC, serum cortisol and late-night salivary cortisol (LNSC); changes in cardiovascular and metabolic-related parameters; blood pressure, waist circumference and weight; changes in physical manifestations of Cushing’s disease; changes in patient-reported outcomes for health-related quality of life; changes in tumor volume; and adverse events. Results were analyzed descriptively; no formal statistical testing was performed.

**Results:**

Of 60 patients who entered, 53 completed the extension, with 29 patients receiving osilodrostat for more than 96 weeks (median osilodrostat duration: 87.1 weeks). The proportion of patients with normalized mUFC observed in the core period was maintained throughout the extension. At their end-of-trial visit, 72.4% of patients had achieved normal mUFC. Substantial reductions in serum cortisol and LNSC were also observed. Improvements in most cardiovascular and metabolic-related parameters, as well as physical manifestations of Cushing’s disease, observed in the core period were maintained or continued to improve in the extension. Osilodrostat was generally well tolerated; the safety profile was consistent with previous reports.

**Conclusion:**

Osilodrostat provided long-term control of cortisol secretion that was associated with sustained improvements in clinical signs and physical manifestations of hypercortisolism. Osilodrostat is an effective long-term treatment for patients with Cushing’s disease.

**Clinical trial registration:**

ClinicalTrials.gov, identifier NCT02180217

## Introduction

Cushing’s disease is a rare but serious disorder resulting from an adrenocorticotropic hormone (ACTH)-producing pituitary adenoma that, in turn, promotes excess adrenal cortisol (1). Chronic exposure to excess cortisol is associated with numerous comorbidities, including hypertension, muscle weakness, hirsutism, central obesity, hypercoagulability and diabetes mellitus, all of which lead to an increased risk of mortality and poor health-related quality of life (HRQoL) (1–3). The longer the exposure to excess cortisol, the lower the chance of reversing morbidity (2).

Although transsphenoidal surgery is the recommended first-line treatment, approximately one-third of patients experience persistent or recurrent disease following surgery (4), and some patients are ineligible for or refuse surgery (4–6). Steroidogenesis inhibitors are usually the first choice for medical treatment (6). The effect of medical treatment can be easily monitored by measurement of serum and urine cortisol. Owing to the unremitting nature of Cushing’s disease, patients often require continued medical therapy to maintain long-term control of cortisol excretion. To date, long-term efficacy and safety data for steroidogenesis inhibitors from prospective clinical trials are limited (7, 8).

Osilodrostat is a potent oral inhibitor of 11β-hydroxylase and is approved for the treatment of adult patients with Cushing’s disease (USA) or endogenous Cushing’s syndrome (EU and Japan) who are eligible for medical therapy (9–12). The LINC 4 study was a multicenter, 48-week, Phase III clinical trial in patients with Cushing’s disease that included an upfront 12-week randomized, double-blind, placebo-controlled period. Osilodrostat led to rapid normalization of mean urinary free cortisol (mUFC) excretion and was significantly superior to placebo at week 12; normal mUFC excretion was sustained in most patients throughout the 48-week core period (13).

Following the 48-week core period, patients could enter an optional open-label extension period intended to run for an additional 48 weeks. Here, we report the long-term efficacy and safety data from the extension of LINC 4. These data augment the existing efficacy and safety profile of osilodrostat (7, 8, 13, 14).

## Methods

### Patients

Eligibility criteria have been described previously (13). Briefly, the study enrolled adult patients with a confirmed diagnosis of persistent or recurrent Cushing’s disease after pituitary surgery and/or irradiation, or *de novo* Cushing’s disease (if not surgical candidates), with mUFC >1.3 times the upper limit of normal (ULN; 138 nmol/24 h or 50 μg/24 h; calculated from three samples collected on three consecutive days, with ≥2 values >1.3 x ULN). Patients who continued to receive clinical benefit from osilodrostat, as assessed by the study investigator, could enter the extension phase.

The study was conducted in accordance with the Declaration of Helsinki, with an independent ethics committee/institutional review board at each site approving the study protocol; patients provided written informed consent to participate and consented again at week 48 to taking part in the extension phase. The trial is registered at ClinicalTrials.gov (NCT02180217).

### Study design

Data from the 48-week core period of this Phase III study, consisting of a 12-week randomized, placebo-controlled, double-blind period followed by a 36-week open-label treatment period, have been published previously (13). The optional open-label extension phase was initially planned to run for an additional 48 weeks (to week 96 for the last patient enrolled). However, patients could continue in the extension only until a managed-access program or alternative treatment became available locally, or until a protocol amendment was approved at their site that specified that patients enrolled in the optional extension phase should come for an end-of-treatment (EOT) visit within 4 weeks or by week 96, whichever occurred first. Patients still receiving clinical benefit from osilodrostat at their EOT visit were eligible to join a separate long-term safety follow-up study (NCT03606408). Consequently, the extension phase ended when all patients had transitioned to the long-term safety follow-up study, if eligible, or had discontinued from the study. Patients continued to receive open-label osilodrostat at the established effective dose from the core phase (dose adjustments were permitted based on efficacy and tolerability; the maximum dose was 30 mg twice daily [bid]).

### Outcomes

Study outcomes assessed during the extension phase were as follows: complete (mUFC ≤ULN), partial (mUFC decrease ≥50% from baseline and >ULN) and mUFC response rate at weeks 60, 72, 84, 96 and 108, then every 24 weeks until the extension EOT visit; change in mUFC, serum cortisol and late-night salivary cortisol (LNSC) at weeks 60, 72, 84, 96 and 108, then every 24 weeks until the extension EOT visit; time to loss of mUFC control, defined as the time (in weeks) from the first collection of post-baseline normal mUFC (≤ULN) to the first mUFC >1.3 x ULN on two consecutive scheduled visits on the highest tolerated dose of osilodrostat and not related to a dose interruption or reduction for safety reasons after week 26; change in cardiovascular/metabolic-related parameters associated with Cushing’s disease (fasting plasma glucose [FPG] and glycated hemoglobin [HbA_1c_]) at weeks 60, 72, 84, 96 and 108, then every 24 weeks until the extension EOT visit; blood pressure, waist circumference and weight every 4 weeks until week 72, then every 12 weeks until week 108, then every 24 weeks until the extension EOT visit; change from baseline in physical manifestations of hypercortisolism at weeks 72, 96 and 108, then every 24 weeks until the extension EOT visit; changes in HRQoL (determined by Cushing’s Quality of Life Questionnaire [CushingQoL] and Beck Depression Inventory II [BDI-II]) at weeks 72 and 96 and the extension EOT visit; and proportion of patients with ≥20% decrease or increase in tumor volume. mUFC (mean of two or three 24-hour urine samples), serum cortisol (measured between 08:00 and 10:00) and LNSC (measured from two samples collected between 22:00 and 23:00) were evaluated using liquid chromatography-tandem mass spectrometry and assessed centrally. Pituitary magnetic resonance imaging with and without gadolinium enhancement was performed locally at weeks 72 and 96 and the extension EOT visit; images were assessed centrally for change in tumor size. Safety was continually assessed from core study baseline throughout the extension for all enrolled patients by monitoring for adverse events (AEs); all AEs from first patient first visit to last patient last visit are reported. AEs of special interest (AESIs) included events related to hypocortisolism, accumulation of adrenal hormone precursors, arrhythmogenic potential and QT prolongation, and enlargement of the pituitary tumor.

### Statistical methods

Analyses presented here are based on cumulative data generated for the full analysis set (all patients enrolled at core study start who received at least one dose of osilodrostat) up to last patient last visit. Safety analyses included all enrolled patients who received at least one dose of osilodrostat and had at least one valid post-baseline safety assessment. All analyses excluded data for patients in the placebo arm collected during the placebo-controlled period. Results were analyzed descriptively, and no formal statistical testing was performed. Correlations were evaluated using the Pearson’s correlation coefficient; extreme outliers were defined as >(Q3 + 3 x IQR) or <(Q1 − 3 x IQR), where Q1 and Q3 are the first and third quartiles and IQR is the interquartile range (Q3 − Q1).

## Results

### Patient disposition and baseline characteristics

**Figure 1:**
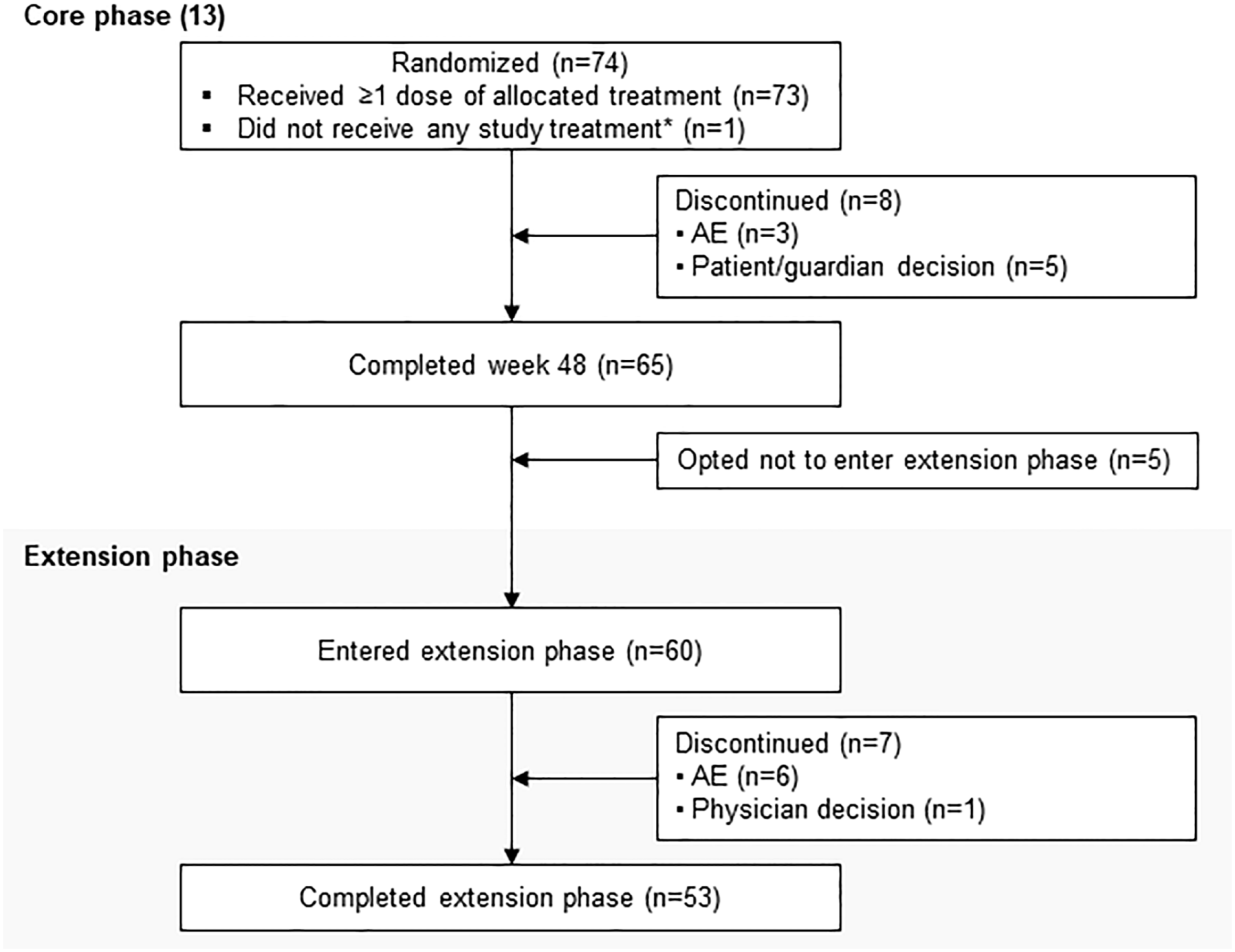
Patient disposition. *Patient was randomly allocated to osilodrostat but did not receive any study treatment because of a serious AE (grade 4 pituitary apoplexy that required hospitalization prior to receiving any study drug) that was not considered related to treatment.

LINC 4 was conducted from October 3, 2016 to December 31, 2020. Of the 73 patients who were enrolled and received treatment in the core phase, 65 completed the core phase and 60 (82.2%) opted to enter the extension; 53 (72.6%) patients completed the extension (**Figure 1**). At core study baseline, most patients had undergone previous pituitary surgery (87.7%) or received prior medical therapy (61.6%; **Table 1**). Patients had a variety of comorbidities at core study baseline, most commonly hypertension (61.6%); physical manifestations of hypercortisolism were common (**Table 1**).

**Table 1:** Core study patient baseline characteristics.

	All patients, N=73
Median age, years (range)	39.0 (19.0–67.0)
Female, n (%)	61 (83.6)
Median time since diagnosis, months (range)	67.4 (6.0–257.7)
Cushing’s disease status, n (%)	
Persistent/recurrent *De novo*	70 (95.9) 3 (4.1)
Previous pituitary surgery, n (%)	64 (87.7)
Previous medical therapy for Cushing’s disease, n (%)	45 (61.6)
Previous pituitary irradiation, n (%)*	9 (12.3)
mUFC, nmol/24 h	
Mean (SD) Median (range)	432 (389) [3.1 x ULN] 340 (21–2607) [2.5 x ULN]
Most common (≥15%) comorbidities, n (%)	
Hypertension Diabetes mellitus**^†^** Osteoporosis Vitamin D deficiency Dyslipidemia Hypothyroidism Fatigue Arthralgia Nephrolithiasis Osteopenia Hypercholesterolemia	45 (61.6) 21 (28.8) 19 (26.0) 18 (24.7) 15 (20.5) 15 (20.5) 13 (17.8) 12 (16.4) 12 (16.4) 12 (16.4) 11 (15.1)
Patients categorized with physical manifestations of hypercortisolism, n (%)	
Central obesity Supraclavicular fat pad Dorsal fat pad Facial rubor Proximal muscle atrophy Hirsutism (females only; n=61) Striae Ecchymoses	63 (86.3) 61 (83.6) 56 (76.7) 46 (63.0) 36 (49.3) 29 (47.5) 30 (41.1) 19 (26.0)

*Patients received stereotactic radiosurgery 2.2–12 years prior to study entry; patients received conventional radiation 3.3–9.4 years prior to study entry. ^†^Includes one case of type 1 diabetes mellitus (n=1). SD, standard deviation.

### Exposure to osilodrostat

**Figure 2:**
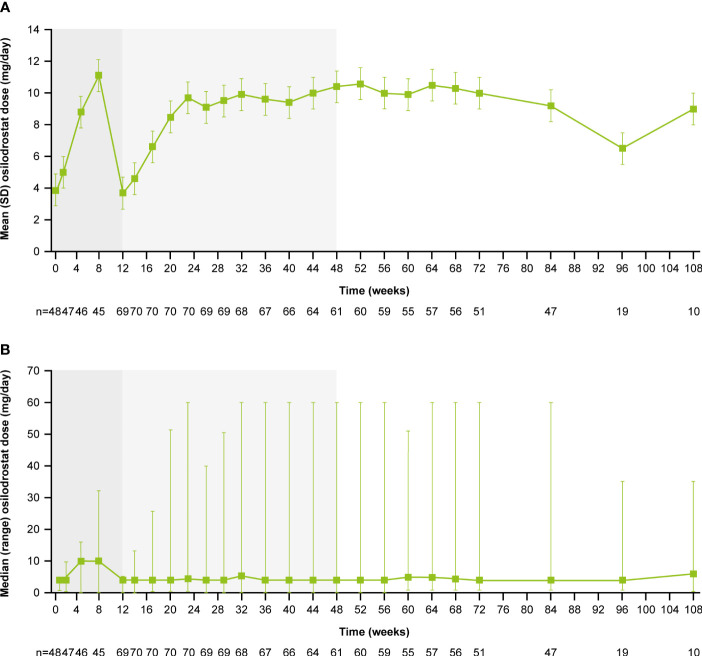
**(A)** Mean and **(B)** median osilodrostat dose over time. Shaded areas indicate the randomized, double-blind period and the open-label period of the core phase. According to the study protocol, all patients restarted the open-label period on osilodrostat 2 mg bid unless they were on a lower dose at week 12. All patients on <2 mg bid osilodrostat (or matched placebo) at week 12 continued to receive the same dose, regardless of initial treatment allocation. n is the number of patients who contributed to the mean/median.

From core baseline to study end, median (range) osilodrostat exposure was 87.1 (2.0–126.6) weeks; 29 (39.7%) patients were exposed to osilodrostat for more than 96 weeks. The median (25^th^–75^th^ percentiles) average osilodrostat dose received during the overall study period was 4.6 (3.7–9.2) mg/day; during the core study, median (25^th^–75^th^ percentiles) average dose was 5.0 (3.8–9.2) mg/day (13). The osilodrostat dose being taken for the longest duration was most frequently 4.0 mg/day (27.4%). Following titration, daily osilodrostat dose remained stable during long-term treatment (**Figure 2**).

### Long-term efficacy of osilodrostat treatment

**Figure 3:**
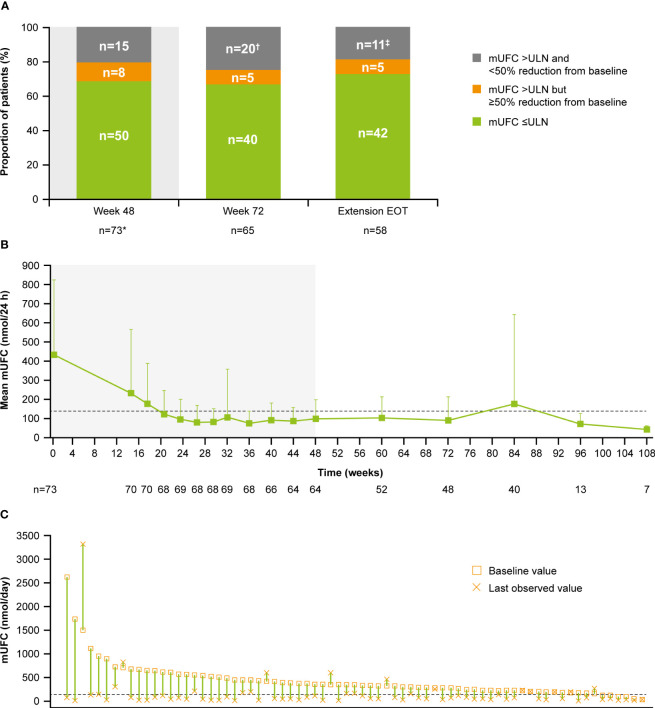
**(A)** Proportion of patients with mUFC response over time, **(B)** mean mUFC over time, and **(C)** individual patient changes in mUFC. **(A)** Patients with missing mUFC at any visit, including those who had discontinued treatment, were counted as non-responders. Shaded area represents the 48-week core phase; excludes data in placebo arm collected during placebo-control period. *The proportion of patients with mUFC ≤ULN at week 48 was calculated using the full analysis set (patients who had discontinued treatment were classified as non-responders). ^†^Discontinued, n=12; missing because of the COVID-19 pandemic, n=4; mUFC not meeting response criteria, n=3; missing (any other reason), n=1. ^‡^mUFC not meeting response criteria, n=8; missing because of the COVID-19 pandemic, n=2; missing (any other reason), n=1. **(B)** Shaded areas indicate the randomized, double-blind period and the open-label period of the core phase. n is the number of patients who contributed to the mean. Analysis includes scheduled visits only. **(B, C)** Dashed line is the ULN for UFC (138 nmol/24 h).

Of patients who had received at least one dose of osilodrostat, 68.5% (n=50/73) had mUFC ≤ULN at the end of the core period, and 54.8% (n=40/73) had mUFC ≤ULN at week 72. Of patients who opted to enter the extension, 66.7% had mUFC ≤ULN (n=40/60) and 8.3% (n=5/60) had mUFC decreased by ≥50% from baseline and >ULN at week 72 (**Figure 3A**). Of patients with an assessment at their extension EOT visit, 72.4% (n=42/58) had mUFC ≤ULN and 8.6% (n=5/58) had mUFC decreased by ≥50% from baseline and >ULN.

Mean mUFC excretion for the 48-week core period of the study has been reported previously (13); mUFC excretion normalized in patients who received osilodrostat, either during the 12-week randomized period (osilodrostat arm) or during the subsequent 36-week open-label period (all patients) (13). Mean mUFC excretion was maintained within the normal range in the extension period (week 72 (n=48), 90.5 [SD 122.6] nmol/24 h; 0.7 [0.9] x ULN; **Figure 3B**). Median (range) mUFC excretion is shown in **Supplementary Figure 1A**. Individual patient changes in mUFC from core study baseline to their last observed visit are shown in **Figure 3C**. There were no escape-from-response events during the extension phase following the primary analysis cut-off (February 25, 2020) (13).

**Figure 4:**
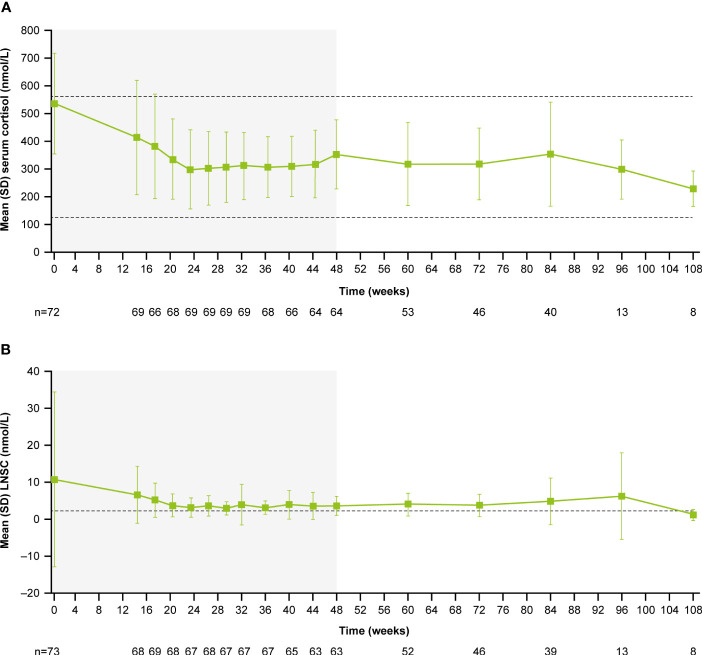
**(A)** Mean serum cortisol and **(B)** mean LNSC from baseline to the end of treatment. Shaded areas indicate the randomized, double-blind period and the open-label period of the core phase. n is the number of patients who contributed to the mean. Dashed line in **(A)** indicates reference serum cortisol range for males and females ≥18 years old (127–567 nmol/L). Dashed line in **(B)** indicates reference LNSC (22:00–23:00) range for males and females ≥18 years old (≤2.5 nmol/L).

During the core period, mean (SD) serum cortisol levels decreased from 538.1 (182.3) nmol/L (0.9 [0.3] x ULN) at baseline to 353.9 (124.9) nmol/L (0.6 [0.2] x ULN) at week 48. Serum cortisol levels then remained stable throughout the extension period (week 72: 319.1 [129.8] nmol/L, 0.6 [0.2] x ULN; **Figure 4A**). LNSC also decreased and then remained stable, although >ULN, throughout the study (baseline: 10.8 [23.5] nmol/L, 4.3 [9.4] x ULN; week 48: 3.7 [2.6] nmol/L, 1.5 [1.0] x ULN; week 72: 3.8 [3.0] nmol/L, 1.5 [1.2] x ULN; **Figure 4B**). Median serum cortisol and LNSC are shown in **Supplementary Figures 1B, C**. Of patients with baseline and last observed value (LOV) measurements, 25.0% had normal LNSC at baseline (n=6/24) and 47.8% had normal LNSC at their last visit (n=11/23). Interpretation of this result is limited by the high degree of missing data (baseline: 67.1%, n=49/73; LOV: 68.5%, n=50/73).

### Changes in cardiovascular and metabolic parameters, physical manifestations of Cushing’s disease and patient-reported outcomes

**Figure 5:**
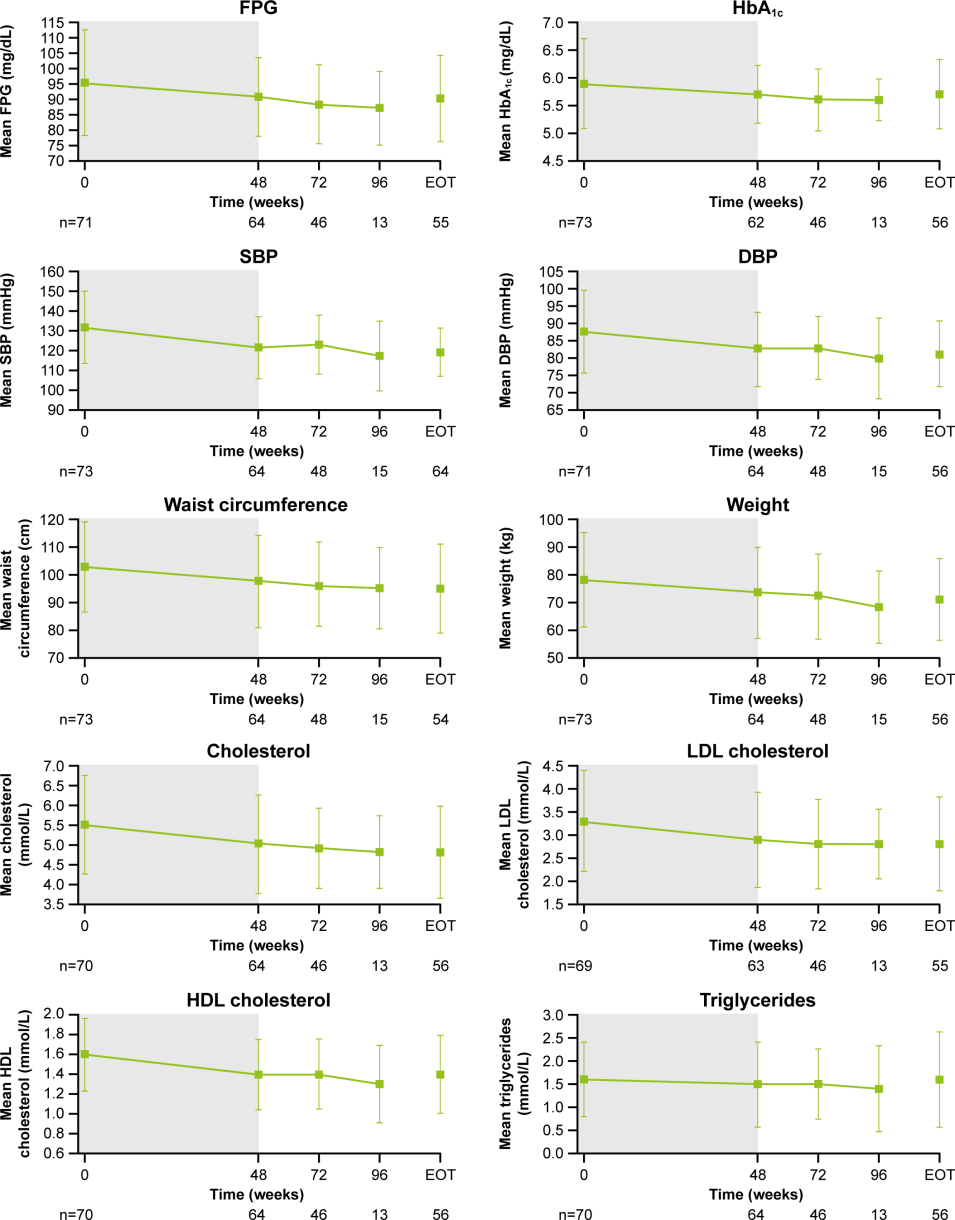
Changes in cardiovascular-related metabolic parameters. Shaded area indicates the core phase. n is the number of patients who contributed to the mean. Error bars indicate standard deviation. DBP, diastolic blood pressure; HDL, high-density lipoprotein; LDL, low-density lipoprotein; SBP, systolic blood pressure.

**Figure 6:**
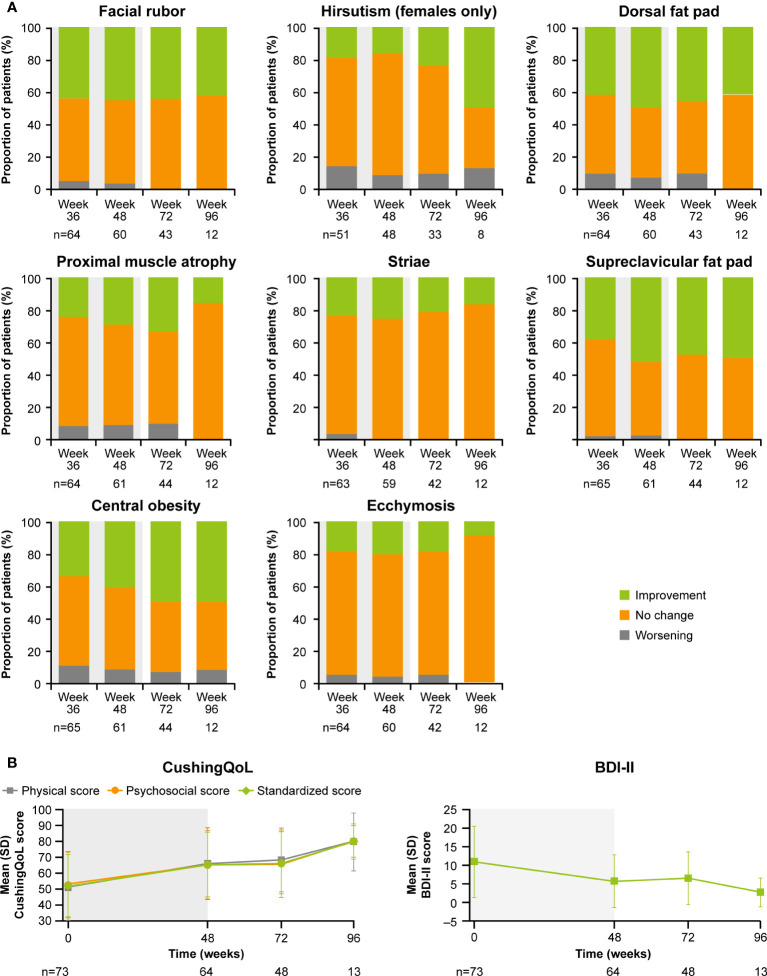
Changes in **(A)** physical manifestations of Cushing’s disease and **(B)** patient-reported outcomes. Shaded area indicates the core phase. n is the number of patients who contributed to the mean.

As previously reported, improvements from baseline occurred in most cardiovascular and metabolic-related parameters in the core period following osilodrostat treatment (9). This trend continued during the extension phase and included a reduction in FPG, HbA_1c_, cholesterol, systolic and diastolic blood pressure, waist circumference, and weight (**Figure 5**). Similarly, the improvements from baseline in physical features of hypercortisolism observed by week 48 were maintained for most parameters throughout the extension (**Figure 6A**), with either no change or improvement observed from baseline in ≥90% patients for all parameters at week 72. Facial rubor, supraclavicular fat pad, dorsal fat pad and central obesity had a favorable shift from baseline in ≥40% of patients at week 72. Few patients reported worsening from baseline of specific manifestations (**Figure 6A**).

Improvements were also observed in scores for patient quality of life (QoL). Both standardized CushingQoL and BDI-II scores improved steadily during the core phase. QoL scores continued to improve further during the extension. At week 72 and EOT, mean (SD) standardized CushingQoL score was 66.4 (19.6) and 69.0 (20.9), and mean (SD) BDI-II score was 6.5 (7.0) and 6.2 (7.1), representing a mean (SD) change from baseline of 15.2 (19.0) and 17.1 (17.1) and −4.1 (9.3) and −4.5 (7.9), respectively (**Figure 6B**).

### Adverse events

AEs that occurred in >20% of patients, irrespective of study-drug relationship, during the entire study period (median [range] osilodrostat exposure for all patients: 87.1 [2.0–126.6] weeks; excluding data collected in the placebo arm during the placebo-controlled period) are shown in **Table 2**. The most common AEs were decreased appetite (46.6%), arthralgia (45.2%) and fatigue (39.7%). Most AEs were mild or moderate; 60.3% were reported as grade 1/2 (**Table 2**).

**Table 2:** Summary of adverse events during LINC 4 core and extension periods.

	All patients* (N=73)	
n (%)	All grades	Grade 3/4
Any AE	72 (98.6)	29 (39.7)
Serious AE	10 (13.7)	9 (12.3)
≥1 AE leading to discontinuation**^†^**	9 (12.3)	2 (2.7)
≥1 AE leading to dose adjustment/interruption	42 (57.5)	13 (17.8)
≥1 AE requiring additional therapy	67 (91.8)	22 (30.1)
Most common AEs (occurring in >20% of patients overall)		
Decreased appetite	34 (46.6)	1 (1.4)
Arthralgia	33 (45.2)	2 (2.7)
Fatigue	29 (39.7)	3 (4.1)
Nausea	27 (37.0)	1 (1.4)
Headache	25 (34.2)	1 (1.4)
Dizziness	22 (30.1)	0
Adrenal insufficiency	19 (26.0)	3 (4.1)
Increased blood testosterone	18 (24.7)	0
Myalgia	18 (24.7)	5 (6.8)
Asthenia	17 (23.3)	2 (2.7)
Diarrhea	17 (23.3)	0
Hypertension	16 (21.9)	9 (12.3)
Upper respiratory tract infection	16 (21.9)	0

A patient with multiple severity grades for an AE is only counted under the maximum grade. *Excludes data collected for placebo recipients during the 12-week randomized period; **^†^** Adrenal insufficiency, n=3; hyperbilirubinemia, hypokalemia, headache, arthralgia, pituitary tumor, benign pituitary tumor, depression, n=1 each (one patient discontinued because of two events, arthralgia and headache).

Overall, 10 AEs (adrenal insufficiency, n=3; hyperbilirubinemia, hypokalemia, headache, arthralgia, pituitary tumor, benign pituitary tumor, and depression, n=1 each) in nine patients (12.3%; one patient experienced both arthralgia and headache) led to treatment discontinuation. For two patients (2.7%), those AEs were reported as grade 3 (hyperbilirubinemia and hypokalemia). One patient discontinued following the primary analysis cut-off date (February 25, 2020).

**Figure 7:**
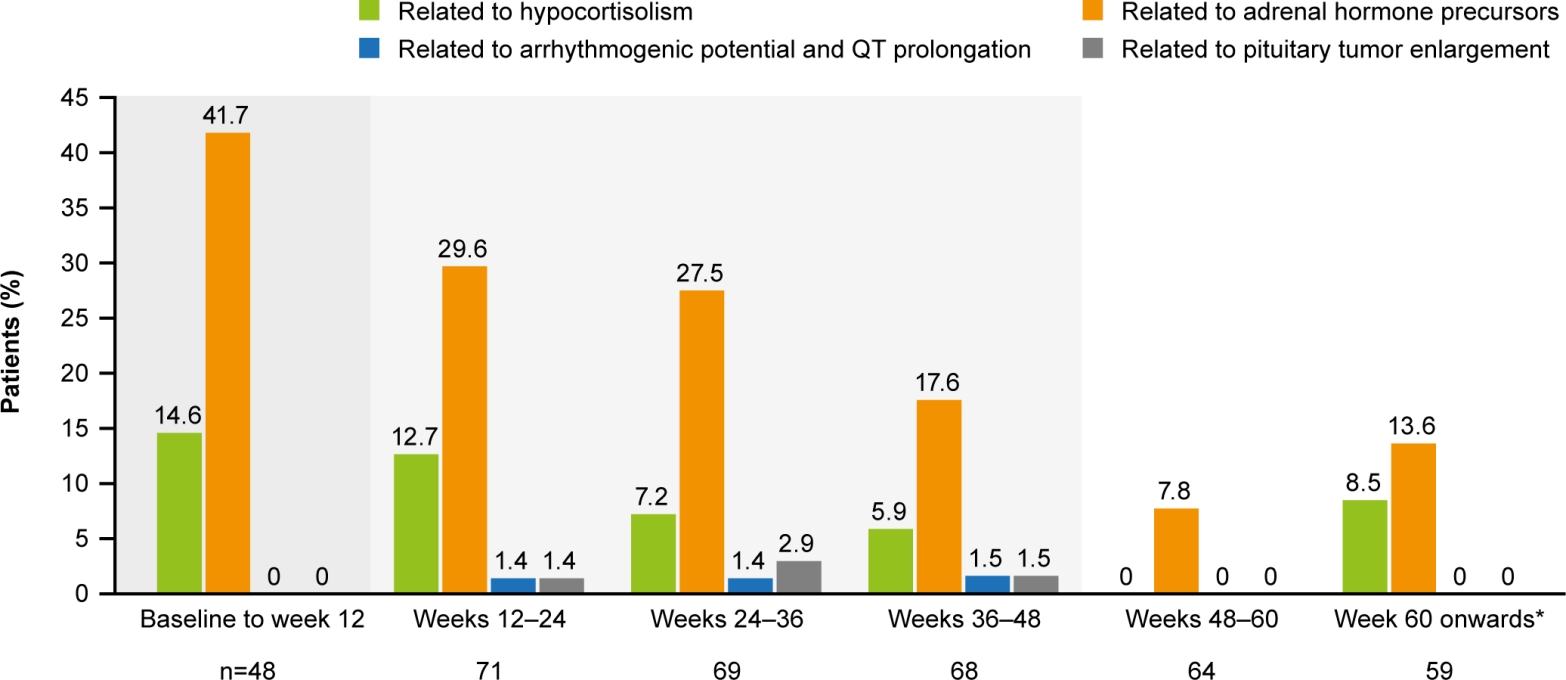
Occurrence of AESIs by time interval. The denominator for each time period only included patients who had at least one scheduled visit, or at least one observed AE, during that period. From baseline to week 12, the denominator only included patients randomized to osilodrostat. A patient with multiple occurrences of an AE within the same period is counted only once in that period. However, if an AE ends and occurs again in a different period, it is then counted in both periods. Shaded areas indicate the randomized, double-blind period and the open-label period of the core phase. *Maximum duration of follow-up was 127 weeks.

The most common AESIs in both the core and extension periods were those related to adrenal hormone precursors. However, the proportion of patients reporting these AESIs was lower in the extension than in the core period (**Figure 7**). AESIs related to hypocortisolism were most frequent during the core period but did occur throughout the remainder of the study, albeit at lower frequency (**Figure 7**). Hypocortisolism-related AEs were most frequently managed with temporary osilodrostat interruption (n=20) or dose adjustment (n=6), and with concomitant glucocorticoids (n=15). There were no new occurrences of AESIs related to arrhythmogenic potential and QT prolongation, or to pituitary tumor enlargement, in the extension (**Figure 7**). During the entire study period from core baseline to the end of the extension, AESIs led to osilodrostat discontinuation in six (8.2%) patients (n=1, related to accumulation of adrenal hormone precursors [hypokalemia]; n=3, related to hypocortisolism [all adrenal insufficiency]; n=2, related to pituitary tumor enlargement [pituitary tumor and pituitary tumor benign]).

**Figure 8:**
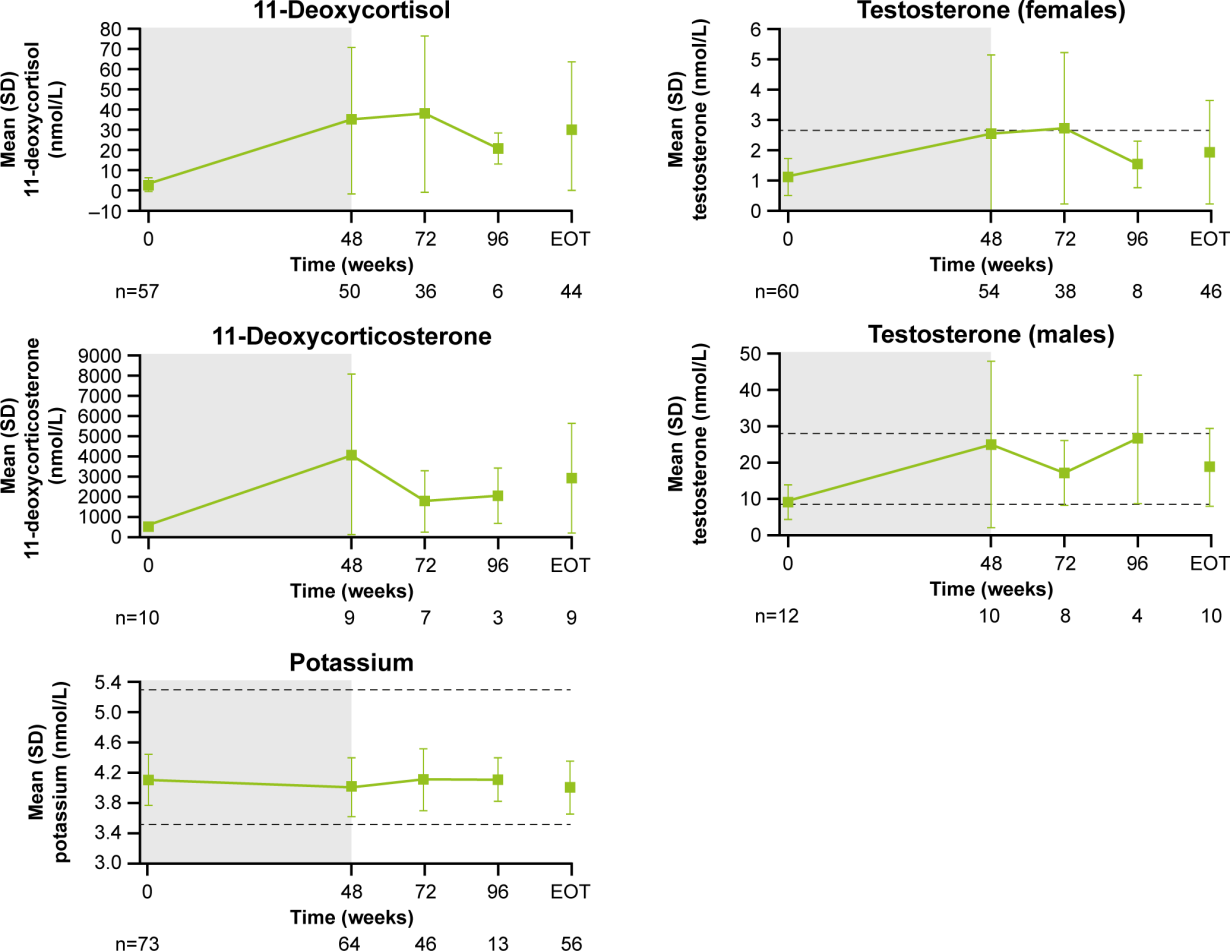
Mean (± SD) levels up to the end-of-treatment visit in the extension phase for 11-deoxycortisol, 11-deoxycorticosterone, potassium and testosterone (in males and females). Shaded area indicates the core phase. n is the number of patients who contributed to the mean. Reference ranges: 11-deoxycortisol ULN, 3.92 nmol/L in males and 3.1 nmol/L in females, or lower depending on age; 11-deoxycorticosterone ULN, 455 pmol/L in males and 696 pmol/L in females (mid-cycle); potassium, 3.5–5.3 mmol/L; testosterone, 8.4–28.7 nmol/L in males and 0.7–2.6 nmol/L in females.

Following an increase in 11-deoxycortisol and 11-deoxycorticosterone during the core study, levels tended to decrease during longer-term treatment (**Figure 8**). From baseline to LOV, the proportion of patients with elevated 11-deoxycorticosterone and 11-deoxycortisol levels increased from 10.0% (n=1/10) to 90.0% (n=9/10) and from 57.9% (n=33/57) to 86.7% (n=5 and 2/60), respectively. In female patients, mean (SD) testosterone levels increased from 1.1 (0.6) nmol/L at baseline to 2.5 (2.6) nmol/L at the end of the core phase, then decreased to within the normal range (0.7−2.6 nmol/L for females) by the extension phase end-of-treatment visit (1.9 [1.7] nmol/L; **Figure 8**). The proportion of females with an elevated testosterone level increased from 15.0% (n=9/61) at baseline to 63.2% (n=24/61) at week 72 and then reduced to 41.7% (n=25/61) at LOV. In males, testosterone levels increased and remained within the normal range throughout osilodrostat treatment (**Figure 8**). The proportion of male patients with testosterone levels below the lower limit of normal decreased from 58.3% (n=7/12) at baseline to 33.3% (n=4/12) at LOV. The proportion of patients experiencing AEs potentially related to increased testosterone (increased blood testosterone, acne and hirsutism) was lower during the extension than during the core study (**Supplementary Figure 2**). Mean serum potassium levels remained stable and within the normal range (3.5–5.3 mmol/L) throughout osilodrostat treatment (**Figure 8**). The proportion of patients with a normal potassium level was similar between baseline (98.6%, n=72/73) and LOV (94.4%, n=68/72).

**Figure 9:**
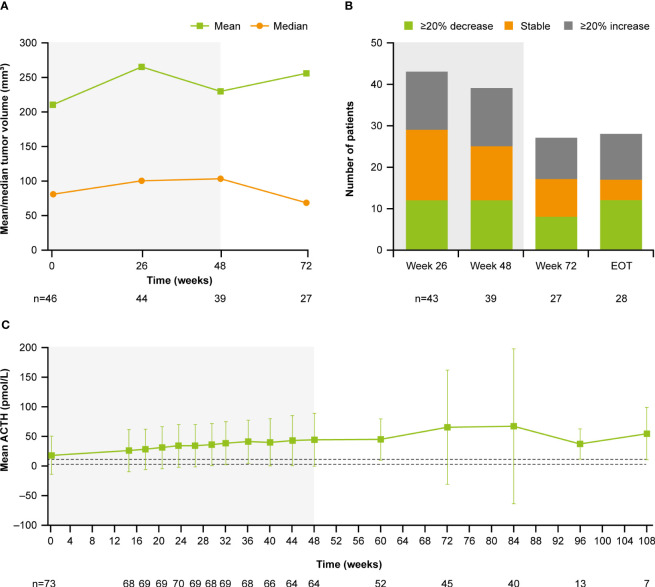
**(A)** Mean and median tumor volume over time, **(B)** number of patients with a change in tumor volume from baseline, and **(C)** mean ACTH over time. Shaded areas indicate the core phase. n is the number of patients who contributed to the mean. Dashed lines in **(C)** indicate reference morning (07:00–10:00) plasma ACTH ranges for males and females ≥18 years old (1.3–11.1 pmol/L).

At baseline, median (range) tumor volume was 82.0 (12.0–2861.0) mm^3^; 28.8% (n=21/73) of patients had a macroadenoma (≥10 mm) and 68.5% (n=51/73) had a microadenoma (<10 mm). At week 72, median (range) tumor volume was 68.0 (10.0–3638.0) mm^3^ (**Figure 9A**). Of the 27 patients with measurements at both baseline and week 72, 29.6% (n=8/27) had a ≥20% decrease in tumor volume and 37.0% (n=10/27) had a ≥20% increase (**Figure 9B**). Notably, mean (SD) plasma ACTH increased steadily between baseline (17.1 [32.1] pmol/L, n=73) and week 72 (65.0 [96.9] pmol/L, n=45; **Figure 9C**); mean ACTH levels appeared to stabilize after week 72. All patients experienced an increase in ACTH levels from baseline to week 72 (n=45) and LOV (n=73); of these, 34/45 (75.6%) and 47/73 (64.4%) experienced an increase in ACTH of ≥2 × baseline levels to week 72 and to LOV, respectively. There was no correlation between change in tumor volume and change in ACTH from baseline to week 72 (*r*=0.1; calculated without two extreme outliers).

## Discussion

Following transsphenoidal surgery, approximately one-third of patients experience persistence or recurrence of disease and subsequently require further treatment to control excess cortisol secretion (4). It is therefore essential that clinical studies evaluating the long-term safety and efficacy of potential new treatments, such as osilodrostat, are performed. The data presented here from the LINC 4 extension reinforce previous reports demonstrating that osilodrostat is effective and well tolerated during long-term treatment of Cushing’s disease (7, 8, 13, 14).

The normalization of mUFC excretion, observed from as early as week 2 in some patients (13), was sustained to the end of the optional open-label extension phase. Overall, the response rate was durable and remained ≥60% throughout the study, with 72.4% of patients maintaining mUFC ≤ULN at their extension EOT visit. Considering the range in baseline mUFC values (21.4–2607.3 nmol/24 h), this indicates that patients can benefit from osilodrostat treatment regardless of their baseline mUFC level. This also suggests that baseline mUFC is not an indicator of whether a patient will respond to osilodrostat treatment. Notably, there were no escape events during the extension period. Additionally, the improvements in most cardiovascular and metabolic parameters, physical manifestations and QoL previously reported during the 48-week core phase were maintained or further improved with long-term treatment (13). Collectively, these results demonstrate the ability of osilodrostat to reduce the burden of disease and comorbidities frequently experienced by patients with Cushing’s disease.

mUFC excretion is commonly assessed in clinical trials and during routine clinical practice to evaluate response to treatment. It is also important to monitor the recovery of the circadian cortisol rhythm in response to treatment by measuring serum cortisol and LNSC (6, 15–17). Elevated LNSC levels have been linked to dysregulation in glucose tolerance, insulin sensitivity and insulin secretion (18). As such, one potential explanation for persistent comorbidities in some patients with normalized mUFC excretion is that LNSC, although reduced, remains just above the ULN. Assessment of LNSC during treatment with other medical therapies has been reported, although differences in treatment duration and patient population type and size limit meaningful comparisons between therapies (15–17). In LINC 4, mean serum cortisol levels remained within the normal range. Mean LNSC improved considerably from baseline but remained above the ULN throughout the study; 47.8% (n=11/23) of patients achieved normalized LNSC at their LOV visit. A numerically large decrease in LNSC, but with mean levels remaining above the ULN, is consistent with previous reports during long-term osilodrostat treatment (8); the mechanism underlying this observation is currently unknown. In real-life clinical practice, the osilodrostat label allows flexible dosing (9, 11), which may help achieve normalization of LNSC. Furthermore, the number of patients with available LNSC assessments was limited, particularly during the extension; therefore, the data should be interpreted with caution. Future studies should examine whether patients with normalization of both UFC and LNSC have better outcomes than patients with only normalized UFC.

Overall, the safety findings reported here for the extension period were consistent with those reported in the primary analysis (13) and previous clinical trials (7, 8, 14). Osilodrostat was generally well tolerated throughout the study; most reported AEs were mild or moderate in severity and manageable. Only nine of 73 (12.3%) patients discontinued osilodrostat at any time because of an AE (3/73 [4.1%] prior to week 48; 6/60 [10.0%] after week 48). Given that osilodrostat is a potent inhibitor of 11β-hydroxylase, AEs related to hypocortisolism or increased levels of adrenal hormone precursors are expected. The frequency of these AEs was lower in the extension period than in the core period, although events did still occur, highlighting the importance of monitoring patients regularly throughout long-term osilodrostat use. AEs potentially related to arrhythmogenic potential and QT prolongation remained infrequent throughout the study. Furthermore, the clinical benefit and tolerability of osilodrostat is supported by the high proportion of patients who chose to continue into the extension period: 92.3% who completed the core phase continued into the optional extension phase, with 88.3% of those completing the extension.

Although dose adjustments were allowed in the open-label phase, the dose of osilodrostat remained stable over long-term treatment, with 4 mg/day adequate for most patients to achieve and sustain control of mUFC excretion. Most AEs related to hypocortisolism occurred during the dose-escalation periods of both LINC 4 (27%) and LINC 3 (51%) (19); the lower occurrence in LINC 4 than LINC 3 may have been related to the more gradual dose-escalation schedule of LINC 4 (every 3 weeks) relative to that of LINC 3 (every 2 weeks) (13, 14, 19). As such, an increased dose-titration interval could be considered when there is a need to mitigate the potential for glucocorticoid withdrawal syndrome or hypocortisolism-related AEs following a rapid decrease in cortisol. Dose-increase decisions should be informed by regular cortisol assessments, the rate of decrease in cortisol, and the individual’s clinical response and tolerability to osilodrostat. Furthermore, as with all steroidogenesis inhibitors, patients should be educated on the expected effects of treatment and dose increases, with a particular focus on the symptoms of hypocortisolism and the advice to contact their physician if they occur.

As expected, levels of 11-deoxycortisol, 11-deoxycorticosterone and, in women, testosterone increased during osilodrostat treatment. These then decreased during long-term treatment; notably, testosterone levels in women returned to within the normal range and to near baseline levels. These observations are consistent with the findings of LINC 3, which also demonstrated that these increases were reversible following discontinuation of osilodrostat (14). Compared with the primary analysis, there were no new AEs of increased testosterone in the extension phase of LINC 4; these findings are consistent with both LINC 2 and LINC 3 long-term analyses (7, 8).

In general, osilodrostat did not adversely affect pituitary tumor volume, with similar proportions of patients reporting either a ≥20% decrease, ≥20% increase or stable tumor volume throughout the study. Although ACTH levels increased during osilodrostat treatment, there was no apparent correlation between the change in ACTH and the change in tumor volume after 72 weeks of treatment; however, longer-term data are needed to evaluate this further. As ACTH-producing pituitary adenomas are the underlying drivers of hypercortisolism, in turn responsible for the high morbidity and poor QoL associated with the disease, tumor stability is of great clinical importance in patients with Cushing’s disease, especially those for whom surgery has failed or is not a viable option.

In addition to LINC 4, other studies have assessed the long-term efficacy and safety of other medical therapies (20–24); however, there is a paucity of prospective, long-term data. For metyrapone, an oral steroidogenesis inhibitor that is given three or four times daily (25), prospective data are currently only available for 36 weeks of treatment in the Phase III/IV PROMPT study (22, 23). Normalization of mUFC excretion was observed in 48.6% (n=17/35) of patients at week 36 (23), and gastrointestinal, fatigue and adrenal insufficiency AEs were the most commonly reported during the first 12 weeks of treatment (22). Current data for levoketoconazole, an oral steroidogenesis inhibitor that is a ketoconazole stereoisomer taken twice daily, are available for 12 months (median duration of exposure 15 months, n=60) following the extended open-label extension of the Phase III SONICS study (26). Of patients with data, 40.9% (n=18/44) had normal mUFC excretion at month 12 (26). During the extension, no patient experienced alanine aminotransferase or aspartate aminotransferase >3 x ULN, suggesting that the potentially clinically important events relating to liver toxicity may be more likely to occur early during treatment, although periodic monitoring during long-term treatment is advisable (26). Pasireotide is a second-generation somatostatin receptor ligand that is administered subcutaneously twice daily (27, 28) or intramuscularly once a month (29–31). In a 12-­month extension of a Phase III study evaluating the long-term efficacy of long-acting pasireotide, 53.1% of patients had normalized mUFC at study completion (median treatment duration 23.9 months), with the most common AEs being related to hyperglycemia (21). The differences in duration and design of these studies prevent a meaningful comparison of the long-term efficacy of medical treatments for Cushing’s disease.

The extension period of LINC 4 was initially planned to run to week 96; however, in agreement with the FDA, a protocol amendment was approved that resulted in approximately half of the patients completing the extension phase between weeks 72 and 96. We also acknowledge the potential for selection bias for patients who experienced the greatest clinical benefit during the 48-week core study; however, over 80% of patients chose to continue osilodrostat treatment after consenting to take part in the extension.

## Conclusions

During the LINC 4 extension period, osilodrostat provided long-term control of cortisol excretion, accompanied by sustained improvements in clinical symptoms, physical manifestations of hypercortisolism and QoL. The safety profile was favorable. These data provide further evidence of the durable clinical benefit of long-term osilodrostat treatment in patients with persistent, recurrent or *de novo* Cushing’s disease.

## Data availability statement

The datasets generated and analyzed during the current study are not publicly available but are available from the corresponding author on reasonable request. Recordati Rare Diseases will share the complete de-identified patient dataset, study protocol, statistical analysis plan, and informed consent form upon request, effective immediately following publication, with no end date.

## Ethics statement

The studies involving human participants were reviewed and approved by an independent ethics committee/institutional review board at each study site. The patients/participants provided their written informed consent to participate in this study.

## Author contributions

The study steering committee (PS, AH, RF, and RA), AP, and the funder designed the study. AH, MG, MB, PW, ZB, AT, and PS enrolled patients in the study. Data were collected by investigators of the LINC 4 Study Group using the funder’s data management systems. MP and the funder’s statistical team analyzed the data. A data-sharing and kick-off meeting was held with all authors and an outline prepared by a professional medical writer based on interpretation provided by the authors. Each new draft of the manuscript subsequently prepared by the medical writer was reviewed and revised in line with direction and feedback from all authors. All authors contributed to the article and approved the submitted version.
