# Supplementary material for: Long-term efficacy and safety of osilodrostat in patients with Cushing’s disease: results from the LINC 4 study extension
Source: Front Endocrinol (Lausanne). 2023 Aug 23;14:1236465. doi: 10.3389/fendo.2023.1236465 (PMC10482037; doi:10.3389/fendo.2023.1236465)
Supplement: Supplementary file 1 [file DataSheet_1.docx]

**Long-term efficacy and safety of osilodrostat in patients with Cushing’s disease: Results from the LINC 4 study extension**

**Supplementary material**

**Supplementary Figure 1. (A)** **Median mUFC, (B) median serum cortisol and (C) median late-night salivary cortisol over time**

**(A)**


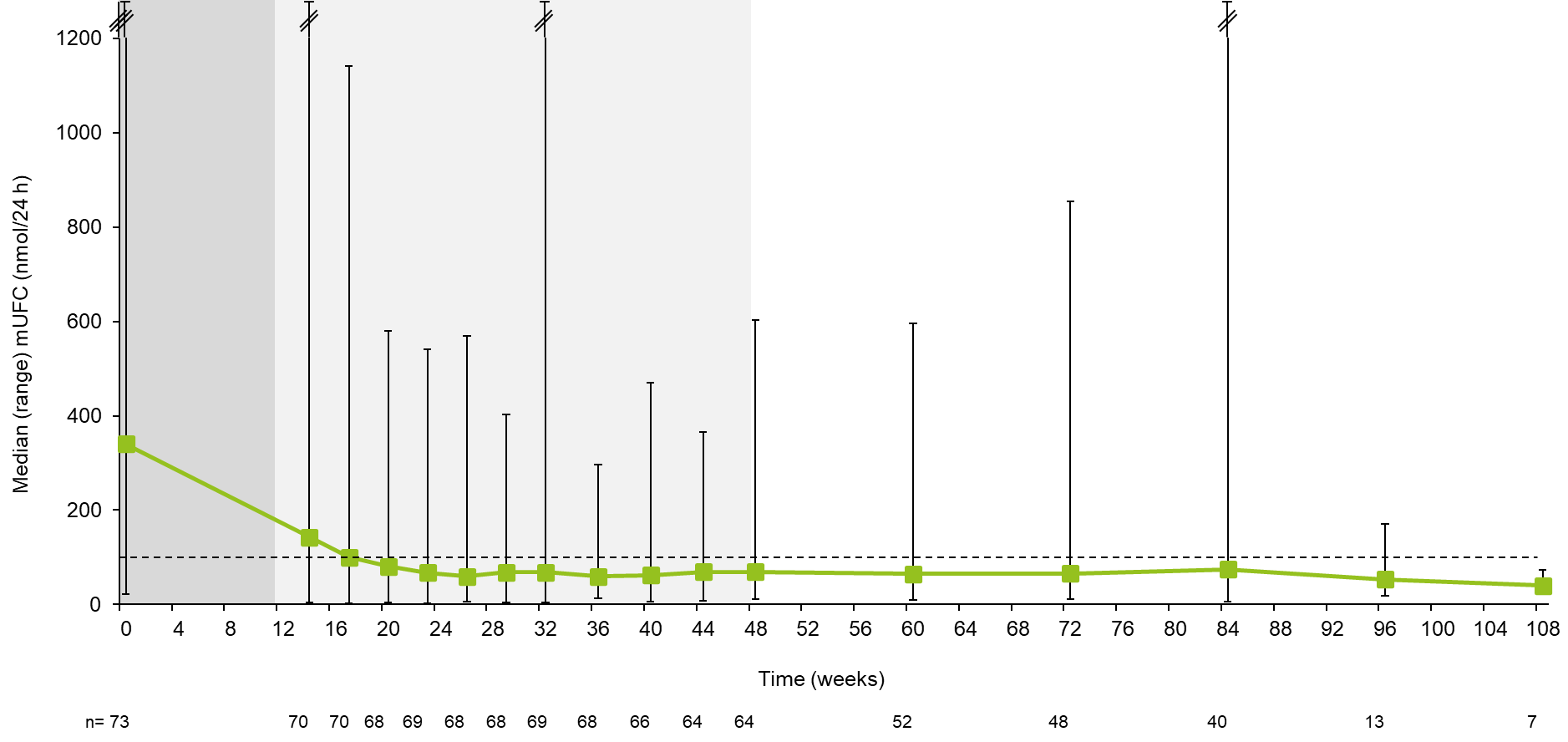


**(B)**


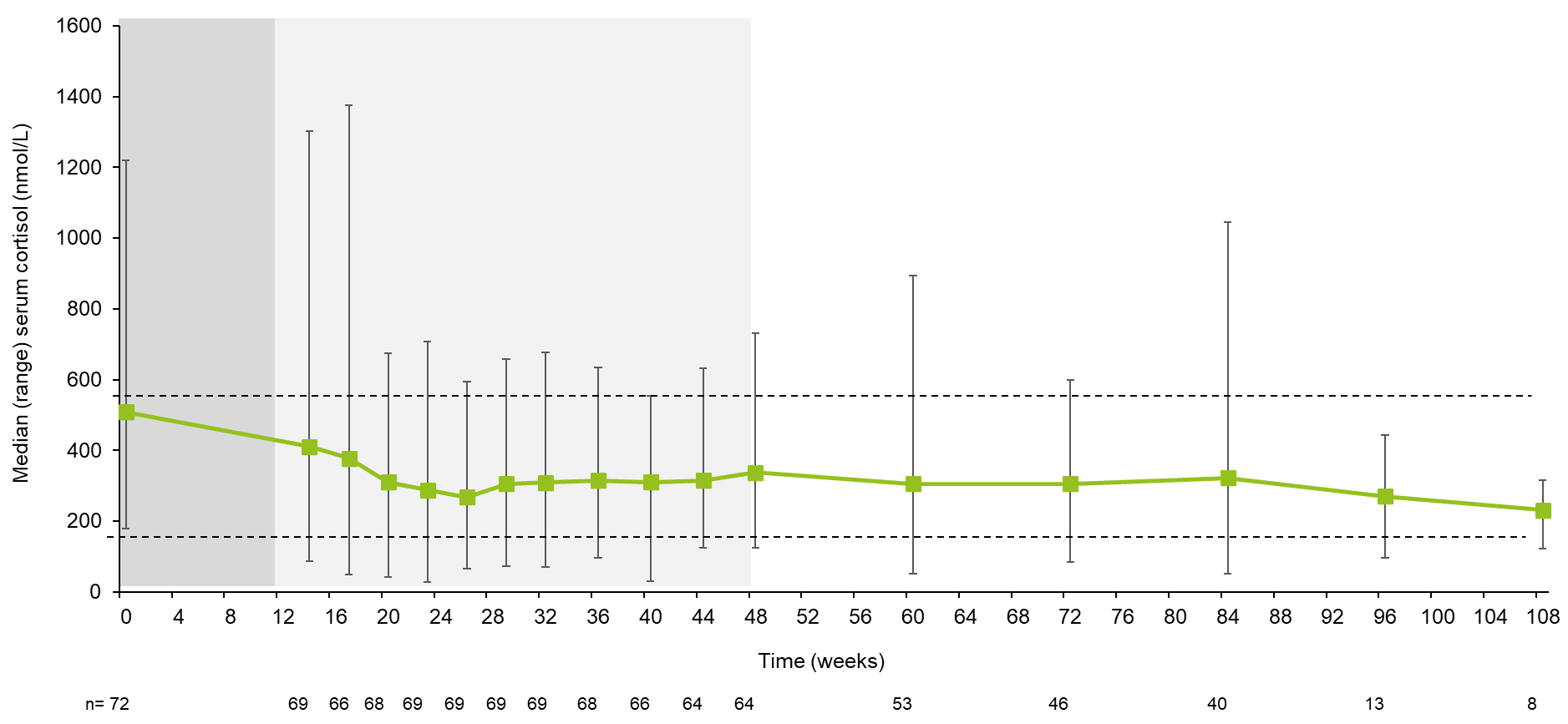


**(C)**


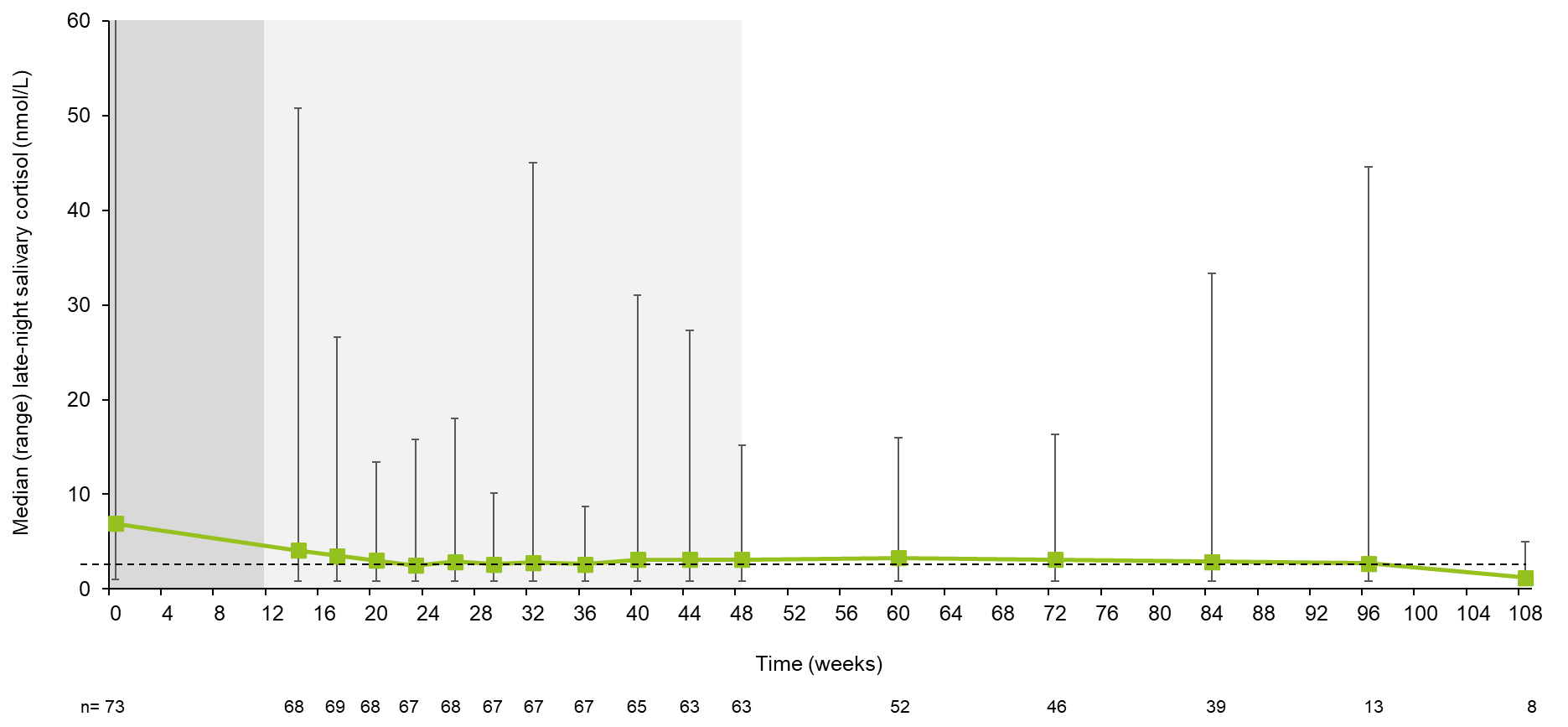


Shaded areas in indicate the randomized, double-blind period and the open-label period of the core phase. n is the number of patients who contributed to the mean. Analysis includes scheduled visits only. Dashed line in A is the ULN for UFC (138 nmol/24 h). Dashed line in (B) indicates reference serum cortisol range for males and females ≥18 years old (127–567 nmol/L). Dashed line in (C) indicates reference LNSC (22:00–23:00) range for males and females ≥18 years old (≤2.5 nmol/L). LNSC, late-night salivary cortisol; mUFC, mean urinary free cortisol; UFC, urinary free cortisol; ULN, upper limit of normal

**Supplementary Figure 2. Occurrence of AEs potentially related to increased testosterone by time interval**

**
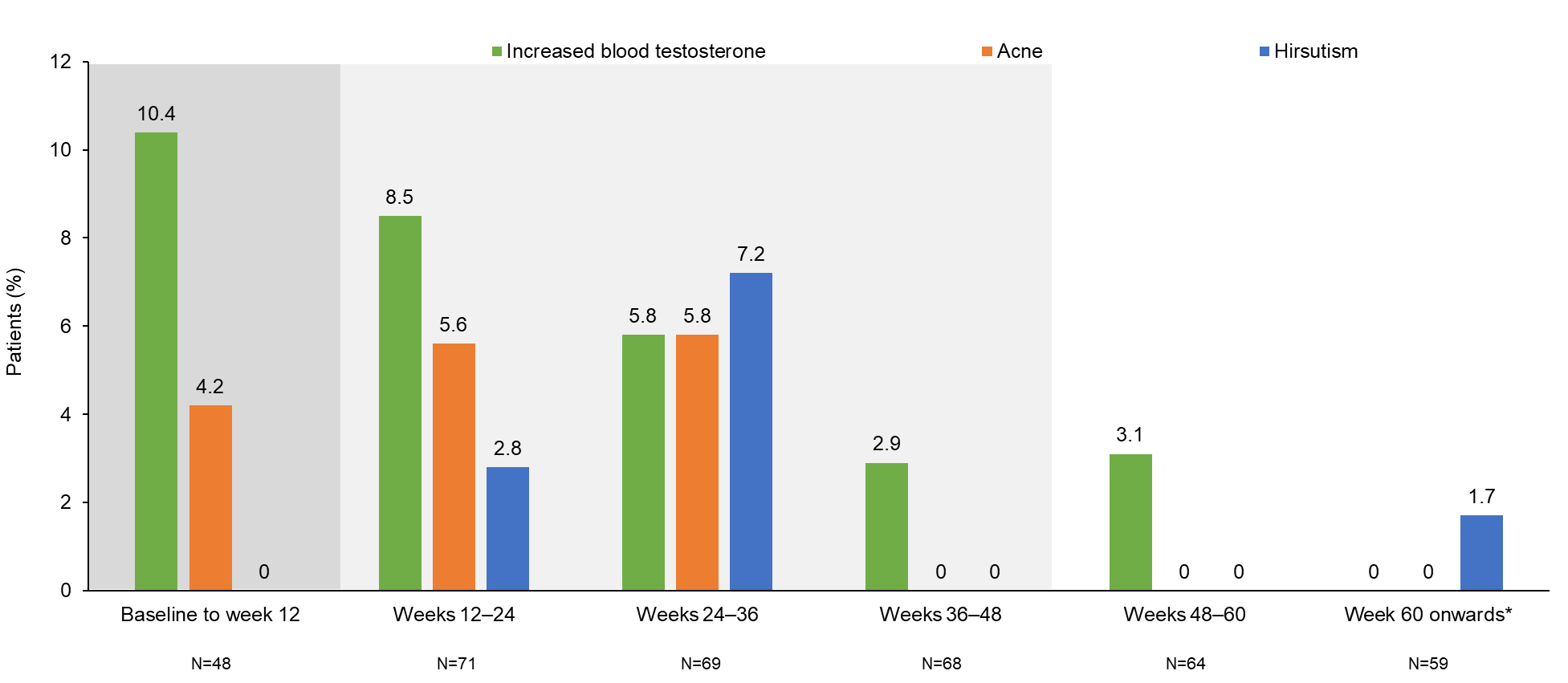
**

The denominator for each time period only included patients who had at least one scheduled visit, or at least one observed AE, during that period. From baseline to week 12, the denominator only included patients randomized to osilodrostat. A patient with multiple occurrences of an AE within the same period is counted only once in that period. However, if an AE ends and occurs again in different period then it is counted in both periods. Shaded areas indicate the randomized, double-blind period and the open-label period of the core phase. *Maximum duration of follow-up was 127 weeks. AE, adverse event
